# Regulation of Surfactant Protein Gene Expression by *Aspergillus fumigatus* in NCl-H441 Cells

**DOI:** 10.3390/microorganisms11041011

**Published:** 2023-04-12

**Authors:** Natalia Schiefermeier-Mach, Lea Heinrich, Lukas Lechner, Susanne Perkhofer

**Affiliations:** Research and Innovation Unit, Health University of Applied Sciences Tyrol/FH Gesundheit Tirol, 6020 Innsbruck, Austria

**Keywords:** *Aspergillus fumigatus*, lung surfactant protein, NCI-H441 cells

## Abstract

*Aspergillus fumigatus* is an opportunistic fungal pathogen that causes serious lung diseases in immunocompromised patients. The lung surfactant produced by alveolar type II and Clara cells in the lungs is an important line of defense against *A. fumigatus*. The surfactant consists of phospholipids and surfactant proteins (SP-A, SP-B, SP-C and SP-D). The binding to SP-A and SP-D proteins leads to the agglutination and neutralization of lung pathogens as well as the modulation of immune responses. SP-B and SP-C proteins are essential for surfactant metabolism and can modulate the local immune response; however, the molecular mechanisms remain unclear. We investigated changes in the SP gene expression in human lung NCI-H441 cells infected with conidia or treated with culture filtrates obtained from *A. fumigatus*. To further identify fungal cell wall components that may affect the expression of SP genes, we examined the effect of different *A. fumigatus* mutant strains, including dihydroxynaphthalene (DHN)-melanin-deficient *ΔpksP*, galactomannan (GM)-deficient *Δugm1* and galactosaminogalactan (GAG)-deficient *Δgt4bc* strains. Our results show that the tested strains alter the mRNA expression of SP, with the most prominent and consistent downregulation of the lung-specific SP-C. Our findings also suggest that secondary metabolites rather than the membrane composition of conidia/hyphae inhibit SP-C mRNA expression in NCI-H441 cells.

## 1. Introduction

*Aspergillus fumigatus* is a saprotrophic fungus and an opportunistic pathogen that produces thousands of small airborne spores (conidia) [1,2]. In immunocompetent persons, *A. fumigatus* conidia are cleared from the lung through different mechanisms including ciliary action of the epithelium and phagocytosis [3,4]. In immunocompromised patients, fungal conidia can survive, germinate and form a vegetative mycelium leading to a wide range of pulmonary diseases such as allergic bronchopulmonary aspergillosis, aspergilloma and even invasive pulmonary aspergillosis [3]. Despite current therapy, the mortality of pulmonary aspergillosis is reported to reach 30% to 80% [5].

The fungal cell wall is responsible for interacting with host cells. On one side, the cell wall provides a primary line of fungal defense against a hostile environment. On the other side, the cell wall components are often targets of the host’s immune system during fungal infections [6]. The cell wall of *A. fumigatus* is composed of polysaccharides (glucans), chitin and galactomannans (GMs) [7,8,9]. The outer layer of dormant *A. fumigatus* conidia has an additional rodlet layer containing hydrophobic RodA proteins and an underlying dihydroxynaphthalene (DHN)-melanin layer [7,10]. The DHN-melanin layer has been revealed to serve as a cover of pathogen-associated molecular patterns (PAMPs), including β-glucan and GM, and further protects fungi against UV irradiation, reactive oxygen species (ROS) and microbial lytic enzymes [11,12]. It was shown that DHN-melanin-deficient mutants produce white hydrophilic spores with an altered surface structure and reduced virulence [12,13,14].

During conidial germination and fungal growth processes, the conidial surface undergoes major changes, the rodlet layer gets shed by aspartic proteases and the protective layer becomes porous, exposing possible PAMPs to the host’s immune system [10,15]. The formation of germ tubes is accompanied by the GM and galactosaminogalactan (GAG) exposure on the surface of *A. fumigatus* hyphae. GAG was shown to enhance the adherence of fungal hyphae to host cells [16,17]. Murine in vivo models demonstrated that GAG-deficient *A. fumigatus* strains do not produce biofilms and are less virulent during invasive aspergillosis [16,18].

Secondary metabolites of *A. fumigatus* such as degradative enzymes and mycotoxins also affect the host–pathogen interaction even though the molecular mechanisms remain largely unknown [19,20]. Culture filtrates obtained from *A. fumigatus* showed suppressive effects on alveolar macrophages and pulmonary leukocytes [21,22]. Extracellular enzymes, e.g., different proteases, cause damage to alveolar epithelial cells by inducing loss of focal contacts followed by cell detachment [23,24]. The treatment of cells with culture filtrates had a strong cytotoxic effect [25,26,27]: it induced cellular shrinkage, desquamation, actin cytoskeleton rearrangement and apoptosis [23,28].

Human lungs have several lines of defense against fungal pathogens. Among them, pulmonary surfactant plays an important role. A pulmonary surfactant is a lipoprotein complex composed of 90% lipids (mainly phospholipids) and 10% surfactant proteins (SP), including SP-A, SP-B, SP-C and SP-D [29]. Surfactant phospholipids decrease the surface tension at the air–water interface in the alveoli during inspiration and prevent alveolar collapse after expiration thus it is essential for gas exchange [30,31]. Furthermore, pulmonary surfactants are crucial for host defense against lung infections. Hydrophilic surfactant-associated proteins, SP-A and SP-D, belong to the family of innate immune proteins, named collectins [32]. SP-A and SP-D can bind surface structures expressed by pathogens and enhance their clearings by immune cells [32]. Hydrophobic proteins, SP-B and SP-C, are secreted along with surfactant phospholipids and are crucial for the surfactant lipid film dynamics [31]. Mutations in human SP-C and SP-B lead to severe chronic respiratory diseases such as chronic interstitial lung disease and idiopathic pulmonary fibrosis [33,34]. SP-B and SP-C were also suggested to be involved in the modulation of the local immune response, however, molecular mechanisms remain unclear [35].

Previous studies showed the impact of lung surfactants on the severity of pulmonary infections. The knockdown of SP-A, SP-C and SP-D genes resulted in significant impairment of host defense in murine models [36,37,38]. SP-C was shown to bind bacterial lipopolysaccharide (LPS) [39]. SP-A and SP-D were demonstrated to directly interact with *A. fumigatus* conidia: the CRD lectin domain of the SP binds sugar residues on the conidial surface and further increases conidia opsonization [8,40,41]. In addition, the binding of the hydrophilic SP led to agglutination of *A. fumigatus* conidia, preventing the dissemination of conidia, thus enhancing phagocytosis by macrophages and neutrophils [8,40]. It was observed that SP-D could directly bind both *A. fumigatus* conidia and hyphae, further inhibiting fungal growth [42].

Previous studies reported that *A. fumigatus* infection results in differential regulation of SP gene expression. Downregulation of the SP-C and SP-D mRNA was observed in human A549 cells infected with *A. fumigatus* conidia [43,44]. Altered gene and protein expressions of SP-A, SP-B, SP-C and SP-D were shown in mice models of *A. fumigatus*-induced allergic airway inflammation [36,45]. Our study aimed to investigate the effect of *A. fumigatus* culture filtrates as well as DHN-melanin-deficient, GAG-deficient and GM-deficient mutant strains of *A. fumigatus* on the mRNA expression of the SP in human lung cells.

## 2. Materials and Methods

### 2.1. Cells

The human lung adenocarcinoma cell line NCI-H441 (ATCC-HTB-174) was obtained from LGC Standards (Wesel, Germany). NCI-H441 cells were cultured in RPMI 1640 medium supplemented with L-glutamine, 10% fetal calf serum (FCS), 100 units/mL penicillin and 100 μg/mL streptomycin, which were all obtained from Capricorn Scientific (Ebsdorfergrund, Germany). For the infection experiments, an antibiotic-free medium was used overnight and during all experiments.

### 2.2. Fungal Strains

*A. fumigatus DAL* (=CBS144-89, [46]), *∆pksP*, *Δugm1* and *∆gt4bc* strains were obtained from V. Aimanianda (Institut Pasteur, Université de Paris, CNRS, Unité de Mycologie Moléculaire, UMR2000, Paris, France). All strains were maintained at 37 °C in 25 cm^2^ culture flasks with filter cap (Biologix, Hallbergmoos, Germany) containing Sabouraud 4% glucose agar (15 g/L agar, 40 g/L D(+)-glucose, 10 g/L peptone; Merck KGaA, Darmstadt, Germany) for 8–10 days. Conidia were collected using a spore buffer (0.9% NaCl (Merck KGaA, Darmstadt, Germany) and 0.01% Tween 80 (Fisher Scientific, Vienna, Austria)). To elude mycelial contamination, the conidial suspension was passed through a cell strainer (40 μm; Thermo Scientific, Vienna, Austria). Conidia swelling was controlled under the microscope, as discussed before [47].

### 2.3. Cell Treatments

Swollen conidia and culture filtrates were prepared as described before [42,47]. In short, to obtain swollen conidia, 10^8^ freshly isolated conidia/mL were incubated in RPMI plus 3.45% MOPS (Fisher Scientific, Vienna, Austria) plus 2% glucose (Merck KGaA, Darmstadt, Germany) on an environmental shaker–incubator ES-20/60 (BioSan, Riga, Latvia) at 37 °C and maintained at 160 rpm for 2 h. Swollen conidia were further diluted for experiments to the desired concentration, as stated below. To generate fungal culture filtrates, 10^8^ conidia/mL were inoculated in 200 mL Sabouraud medium (40 g/L D(+)-glucose (Merck KGaA, Darmstadt, Germany), 10 g/L peptone from casein (Carl Roth, Karlsruhe, Germany)) and kept on an environmental shaker–incubator ES-20/60 (BioSan, Riga, Latvia) for 24 h at 37 °C and 160 rpm. The suspension was further filtered using a sterile Whatman^®^ paper filter (Merck KGaA, Darmstadt, Germany), and mycelia were washed with double-distilled water, resuspended and incubated in 200 mL cell culture medium for 24 h at 37 °C and 160 rpm. The sterile culture filtrate medium was incubated in parallel as a control. After 24 h, the culture filtrate suspension was sterile-filtered with a 500 mL Nalgene™ Rapid-Flow™ sterile disposable filter unit with 0.2 µm PES membrane (ThermoScientific, Vienna, Austria) and stored in sterile 15 mL tubes (Corning GmbH, Kaiserslautern, Germany) at −20 °C.

For infection experiments, NCI-H441 cells were seeded into 6-well plates (Biologix, Hallbergmoos, Germany) with 10^6^ cells per well and cultured for 26–28 h. Before infection with *A. fumigatus* conidia, cells were washed with serum- and antibiotic-free medium and incubated in this medium overnight. Then, 10^7^ swollen conidia/mL were directly applied to the cells and incubated for 4 and 8 h at 37 °C and 5% CO_2_. Untreated cells were incubated in parallel as a control in each experiment. For the experiments with culture filtrates, filtrates diluted 1:5 in serum-free medium were applied on cells in 6-well plates (10^6^ cells per well) and incubated for 4 and 8 h at 37 °C and 5% CO_2_. Cells treated with a control filtrate medium in the same dilution were incubated in parallel as a control.

### 2.4. Microscopy

The kinetics of the hyphal growth was examined by time-lapse video microscopy, as previously described [42]. In brief, 10^5^ swollen conidia/mL were transferred to the 8-well Nunc™ Lab-Tek™ chamber slide system (Thermo Scientific, Vienna, Austria), and live imaging was performed in a cell incubator at 37 °C and 5% CO_2_ using an ioLight Portable Microscope (ioLight, Hampshire, UK) operated with the ioLight app (Vers.: 1.1.1.483) at 37 °C. Video sequences, tracking and quantification of the velocity of hyphal growth were prepared using Fiji software [48] and the “Manual Tracking” plug-in (developed at Institute Curie by F. Cordeli for ImageJ; Orsay, France). Figures were prepared using Fiji software (based on ImageJ 1.51n, maintained by the Laboratory for Optical and Computational Instrumentation at the University of Wisconsin-Madison, Madison, WI, USA) and Adobe Photoshop (Adobe Systems Incorporated, San José, CA, USA).

### 2.5. Real-Time qPCR

RNA was isolated using Tri Reagent™ Solution (Invitrogen, Vienna, Austria) according to the manufacturer’s instructions. RNA concentration was measured in duplicates using a NanoPhotometer NP80 (Implen, Munich, Germany). cDNA was prepared using the RevertAid First Strand cDNA Synthesis Kit (Thermo Scientific, Vienna, Austria). TaqMan gene expression master mix and TaqMan gene expression assays were used.

Two genes, *SP-A1* and *SP-A2*, encode SP-A1 and SP-A2 proteins in humans [49], and the following assays were used in our experiments: *SP-A1* (Hs00831305_s1), *SP-A2* (Hs04195463_g1), *SP-B* (Hs01090667_m1), *SP-C* (Hs00953663_g1) and *SP-D* (Hs01108490_m1), which were from Thermo Fisher Scientific (Vienna, Austria). *RPLP0* (Hs99999902_m1) was used as the reference gene. For *SP-A1*, *SP-A2*, *SP-B* and *RPLP0*, RNA concentration of 50 ng was used, and for SP-C and SP-D, -100 ng was used for cDNA synthesis. The qPCR reaction was performed in 96-well plates (Biozym, Vienna, Austria). The following run method was used: Step 1, at 50 °C for 2 min; Step 2, at 98 °C for 10 min; Step 3, at 98 °C at 25 s; and Step 4, at 60 °C for 1 min. Steps 3 and 4 were repeated 40 times. (StepOnePlus RT-PCR cycler; Applied Biosystems, Vienna, Austria). Data obtained were calculated using the 2^−ΔΔCT^ method to analyze relative changes in gene expression. As a calibrator for the 2^−ΔΔCT^ method, the untreated NCI-H441 cells were used.

### 2.6. Statistics

All probes were isolated in three independent experiments with two technical repetitions; each experiment included an untreated control performed in the same experiment in parallel. For RT-qPCR experiments, technical duplicates were used for each probe. Data were further analyzed using a multiple unpaired T-test in GraphPad Prisma 9 (GraphPad Software, Boston, MA, USA). Only results with a *p*-value ≤ 0.05 were considered significant. RT-qPCR data were represented as fold change in gene expression normalized to *RPLP0* rRNA expression and relative to untreated NCI-H441 cells (taken as one for each single biological and technical repetition).

## 3. Results

### 3.1. Kinetics of Fungal Growth

**Figure 1:**
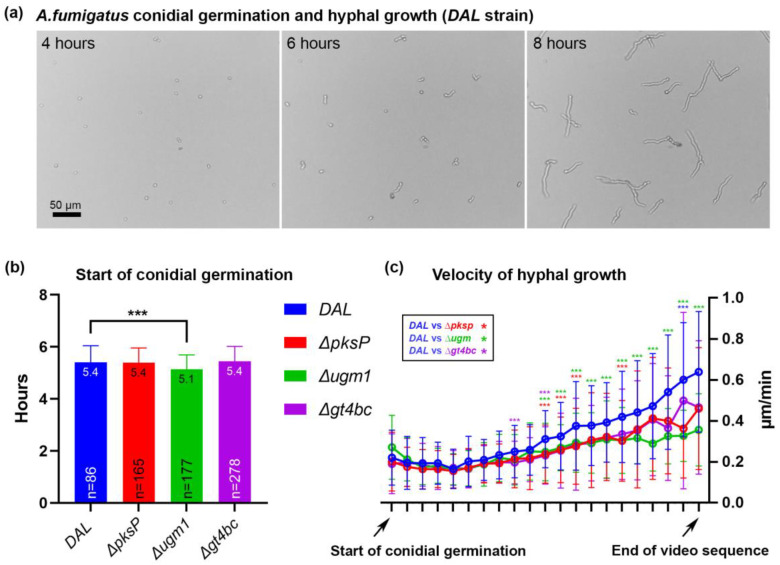
Conidial germination and dynamics of hyphal growth. (**a**) Frames of a live-video sequence show examples of hyphal growth after 4, 6 and 8 h of imaging; (**b**) starting point of conidial germination in four tested *A. fumigatus* strains. Depicted are mean ± SD of mean as a column, mean as value and number of quantified conidia, n; (**c**) velocity of hyphal growth. For graphical representation, all germinating conidia were plotted to the first tick of the x-axis with 10 min between data points. Each tick depicts velocity mean ± SD of mean in µm/min. In (**b**,**c**) significant differences (*p* ≤ 0.001) between *A. fumigatus* strains are marked with *** according to the color scheme.

To exclude significant differences in fungal growth between the four *A. fumigatus* strains and better define time points for the infection experiments, we quantified conidial germination and hyphal growth dynamics and compared *ΔpksP*, *Δugm1* and *Δgt4bc* to the *DAL* strain using time-lapse video microscopy. A total of 10^5^ mL pre-swollen conidia were plated on LapTeck slides followed by imaging for 10 h. The start of conidial germination as well as the velocity of hyphal growth (defined as changes in hyphal length per minute) were quantified. Conidial germination started shortly after 5 h of imaging, and a small delay in gemination was detected in *Δugm1* strain conidia (Figure 1a,b).

When we further tracked hyphal growth in individual conidia and quantified the velocity of hyphae growth, we observed that in the first 2 h after the start of germination, no significant differences in growth velocity were observed when comparing *DAL* (0.21 µm/min ± 0.03 SD) with *ΔpksP* (0.19 µm/min ± 0.02), *Δugm1* (0.20 µm/min ± 0.04) and *Δgt4bc* (0.19 µm/min ± 0.01) strains. At later time points, the velocity of growth increased exponentially in all strains and reached 0.64 µm/min ± 0.29 (*DAL*), 0.46 µm/min ± 0.30 (*ΔpksP*), 0.36 µm/min ± 0.18 *(Δugm1*) and 0.47 µm/min ± 0.33 (*Δgt4bc*) at the end of the video sequence. We observed a slightly increased velocity at several time points when comparing the growth dynamics of *DAL* with *ΔpksP*, *Δugm1* and *Δgt4bc* strains (marked with asterisks in Figure 1c); the most prominent difference was observed between the *DAL* and *Δugm1* strain (green asterisks in Figure 1c). This observation is in line with previous studies showing a reduced growth phenotype for the *Δugm1* mutant [50]. Based on the kinetics of fungal growth in our experiments, we defined two time points of special interest for the following RT-qPCR experiments: 4 h (conidia not yet germinated in any of strains) and 8 h (developed germ tubes in all tested strains).

### 3.2. Infection of Human NCI-H441 Cells with A. fumigatus Conidia

The lung adenocarcinoma human NCI-H441 cell line resembles bronchiolar epithelial Clara cells in phenotype. It was previously used to evaluate the protein and gene expression of SP [51,52]. In line with previous studies, we detected high levels of *SP-A1*, *SP-A2* (Ct values < 19 in control samples) and *SP-B* (Ct < 23) and low levels of *SP-C* (Ct < 30) and SP-D (Ct < 29) gene transcripts [51].

**Figure 2:**
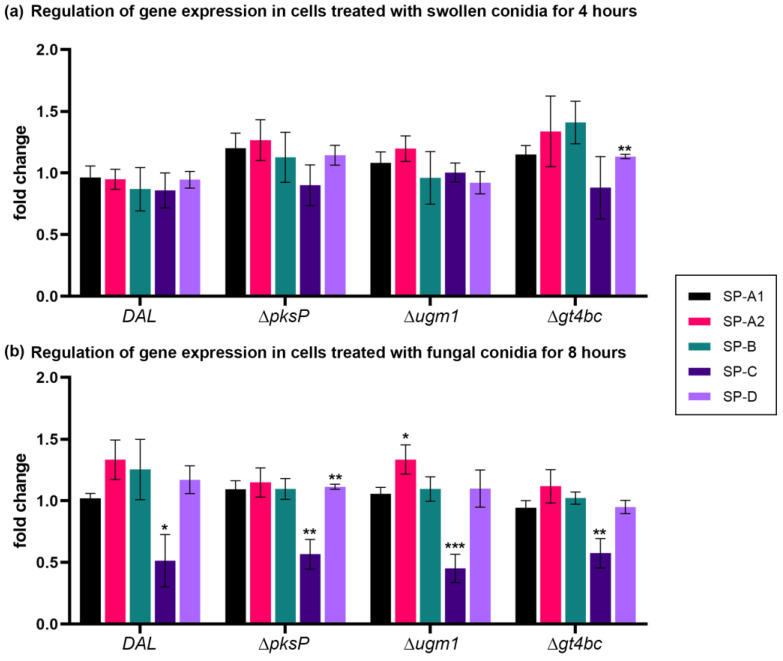
Relative fold change of the mRNA expression levels of *SP-A1*, *SP-A2*, *SP-B*, *SP-C* and *SP–D*. All data are normalized with *RPLP0* rRNA expression and given as relative to untreated control. Cells were treated with swollen conidia obtained from *A.fumigatus* strains for 4 h (**a**) and 8 h (**b**). Columns depict mean ± SE of the mean (* *p* ≤ 0.05), (** *p* ≤ 0.01), (*** *p* ≤ 0.001). See also Table 1.

Next, the relative gene expression of SP in cells separately infected with four *A. fumigatus* strains was measured (Figure 2). When cells were infected for 4 h, no significant changes were observed, as compared to the untreated controls, except for a slight elevation in the *SP-D* gene expression after infection with *Δgt4bc* strain conidia. However, when cells were infected for 8 h, a significant downregulation of the *SP-C* expression was observed in cells treated with *DAL* (fold change: 0.51 ± 0.19, *p* < 0.05), *ΔpksP* (0.57 ± 0.11, *p* < 0.01), *Δugm1* (0.45 ± 0.10, *p* < 0.001) and *Δgt4bc* strain (0.58 ± 0.11, *p* < 0.01) conidia. Interestingly, the fungal infection had no significant effect on the *SP-A1* and *SP-D* expression, except for a slight upregulation of *SP-A2* by *Δugm1* (fold change: 1.34 ± 0.12, *p* < 0.05,) and *SP-D* by the *ΔpksP* (fold change: 1.11 ± 0.02, *p* < 0.01). No significant differences were detected in the other samples (Figure 2, Table 1).

### 3.3. Treatment of Human NCI-H441 Cells with A. fumigatus Culture Filtrates

Next, NCI-H441 cells were treated with culture filtrates collected from the *DAL A. fumigatus* strain. The treatment of cells with undiluted culture filtrates or 1:3 dilution with growth medium caused immediate cell detachment (data not shown). Furthermore, 1:5 dilution of filtrates in the growth medium was well tolerated by cells and, therefore, used in our experiments.

**Figure 3:**
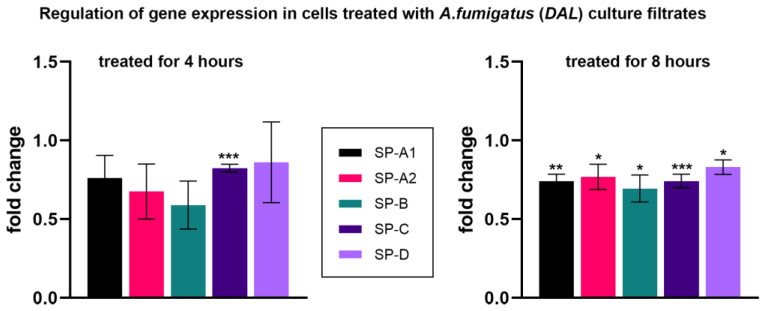
Relative fold change of the mRNA expression levels of *SP-A1*, *SP-A2*, *SP-B*, *SP-C* and *SP–D*. All data are normalized with *RPLP0* rRNA expression and given as relative to untreated control. Cells were treated with culture filtrates obtained from *A. fumigatus* DAL strain for 4 h (left graph) and 8 h (right graph). Columns depict mean ± SE of the mean (* *p* ≤ 0.05), (** *p* ≤ 0.01), (*** *p* ≤ 0.001). See also Table 1.

After 4 h of treatment with culture filtrates, a small downregulation of the *SP-C* gene expression was observed (0.82 ± 0.03, *p*-value < 0.001), while *SP-A1*, *SP-A2*, *SP-B* and *SP–D* mRNA were not regulated. However, treatment with culture filtrates for 8 h resulted in a moderate downregulation of all SP mRNA, with SP-C being the most significant (Figure 3, Table 1).

## 4. Discussion

In this study, we evaluated the gene expression of the pulmonary SP (*SP-A1*, *SP-A2*, *SP-B*, *SP-C* and *SP-D*) in human NCI-H441 cells following an *A. fumigatus* infection. We tested the effect of DAL, DHN-melanin-deficient, GAG-deficient and GM-deficient mutant *A. fumigatus* strains as well as culture filtrates incubated with cells for 4 and 8 h.

Our results show that a short-term infection of NCI-H441 cells did not regulate the SP mRNA. Instead, 8 h of infection caused consistent downregulation of the *SP-C* gene. Within this time, conidial attachment, germination and hyphal growth took place [47,53]. Thus, in comparison to short-term experiments with conidia, NCI-H441 cells were exposed to another biochemical composition of the fungal cell wall when PAMPs, e.g., β-glucan, chitin, GM and GAG, were exposed to lung cells [10,15]. Surprisingly, we did not observe a significant downregulation of *SP-A* and *SP-D* mRNA and no difference was identified between infections with DHN-melanin-, GAG- and GM-deficient strains.

Previous studies demonstrated that infection with *A. fumigatus* caused the differential regulation of surfactant gene expression in vitro and in vivo. In line with our results, a downregulation of lung-specific *SP-C* mRNA was observed in cells and mouse models [36,43,44]. A study of *A. fumigatus*-induced allergic airway inflammation in mice showed decreased mRNA and protein expression of SP-B and SP-C that was accompanied by an upregulated SP-D protein expression without changes in mRNA levels [36]. Protein and mRNA levels of SP-A were not changed in this model [36]. Surprisingly, we did not identify significant differences between the four mutant strains: the downregulation of *SP-C* mRNA was independent of the fungal strain. A more recent report revealed the downregulation of *SP-D* mRNA in human alveolar A549 cells after 6 h of incubation with *A. fumigatus* conidia [44]. In our preliminary experiments, we tested a long-term culture model of A549 cells as a model to consider the deferential regulation of SP in infection settings. This model was shown to express increased levels of SP, as compared to regular A549 cell cultures [54]. Infection of these cells with *A. fumigatus* swollen conidia did not cause any consistent changes in *SP-A* and *SP-D* mRNA in our experiments (unpublished data).

Our data suggest that secondary metabolites of *A. fumigatus*, rather than contact/binding to the fungal cell wall polysaccharides, may alter the mRNA expression of SP. In line with this hypothesis, we observed that the treatment of NCI-H441 cells with culture filtrates resulted in a moderate decrease in gene expression of all SP mRNA when cells were incubated for 8 h. Short exposure to culture filtrates caused a small inhibition of *SP-C* mRNA, whereas changes in other SP genes were not statistically significant. Culture filtrates gained from *A. fumigatus* were previously shown to have a cytotoxic effect on alveolar cells [55] and inhibit a pulmonary immune response [21,56,57]. The effect of the filtrates on SP expression has not been reported before. Importantly, we used diluted filtrates that did not cause cell detachment or apoptosis and observed a moderate inhibiting effect on all tested SP genes.

The major function of SP-C is to reduce the surface tension at the air–liquid alveolar interface by regulating lipid adsorption and transfer of lipids between surfactant membranes, thus providing surfactant film stability [58]. Data from patients and mice models showed that SP-C has an important antimicrobial and anti-inflammatory function in the lung. Thus, patients with mutations in the *SP-C* gene developed familial interstitial lung disease [34]. Knockout of the *SP-C* gene in mice resulted in progressive lung inflammation [59]. These mice were also more susceptible to respiratory syncytial virus and *Pseudomonas aeruginosa* and showed robust inflammation in the lungs after gram-negative bacterial infection [37,60]. The binding of SP-C to bacterial lipopolysaccharide has been observed in vitro [35,59,61]. Several studies showed that exogenous synthetic or natural surfactant treatment reduced symptoms of lung infection and improved bacterial clearance [62]. In vitro experiments demonstrated that treatment with Survanta (a natural surfactant product containing SP-B and SP-C and phospholipids) inhibited proinflammatory cytokine release from LPS-stimulated human alveolar macrophages [63]. Thus, decreased levels of SP-C may impair the resolution of lung inflammation and stimulate progressive interstitial disease [59]. The molecular mechanisms of *SP-C* inhibition by *A. fumigatus* observed in our study and by others remain unclear.

To summarize, our data show consistent downregulation of lung-specific *SP-C* mRNA in NCI-H441 cells caused by *A. fumigatus*. Further elucidating this inhibitory effect in more detail is important to better understand how potentially infectious pathogens overcome the defense mechanisms in the human lung. Moreover, data on the combinational use of pulmonary surfactants and antifungal agents against *A.fumigatus* have only started to arise [42,64] and should be investigated in future studies.

## Figures and Tables

**Table 1:** Statistical analysis of RT-qPCR data.

NCI-H441 cells infected with *A. fumigatus* conidia for 4 h										
	*SP-A1*		*SP-A2*		*SP-B*		*SP-C*		*SP-D*	
Strains	Mean ± SE	*p*-value	Mean ± SE	*p*-value	Mean ± SE	*p*-value	Mean ±SE	*p*-value	Mean ± SE	*p*-value
*DAL* vs. control	0.96 ± 0.10	0.706	0.95 ± 0.08	0.557	0.87 ± 0.18	0.496	0.86 ± 0.10	0.218	0.95 ± 0.07	0.464
*ΔpksP* vs. control	1.20 ± 0.12	0.170	1.27 ± 0.17	0.185	1.13 ± 0.20	0.567	0.90 ± 0.12	0.439	1.14 ± 0.08	0.146
*Δugm1* vs. control	1.08 ± 0.09	0.403	1.20 ± 0.10	0.130	0.96 ± 0.21	0.862	1.00 ± 0.06	0.966	0.92 ± 0.09	0.428
*Δgt4bc* vs. control	1.15 ± 0.07	0.115	1.34 ± 0.29	0.304	1.41 ± 0.17	0.076	0.88 ± 0.18	0.538	1.13 ± 0.02	** 0.002
NCI-H441 cells infected with *A. fumigatus* conidia for 8 h										
	*SP-A1*		*SP-A2*		*SP-B*		*SP-C*		*SP-D*	
Strains	Mean ± SE	*p*-value	Mean ± SE	*p*-value	Mean ± SE	*p*-value	Mean ± SE	*p*- value	Mean ± SE	*p*-value
*DAL* vs. control	1.02 ± 0.04	0.691	1.33 ± 0.16	0.105	1.25 ± 0.25	0.359	0.51 ± 0.21	* 0.051	1.17 ± 0.11	0.206
*ΔpksP* vs. control	1.09 ± 0.07	0.263	1.15 ± 0.12	0.279	1.10 ± 0.09	0.323	0.57 ± 0.12	** 0.007	1.11 ± 0.02	** 0.005
*Δugm1* vs. control	1.06 ± 0.05	0.356	1.34 ± 0.12	* 0.047	1.10 ± 0.10	0.395	0.45 ± 0.12	*** < 0.001	1.10 ± 0.15	0.550
*Δgt4bc* vs. control	0.94 ± 0.06	0.380	1.12 ± 0.14	0.435	1.02 ± 0.05	0.689	0.58 ± 0.12	** 0.007	0.95 ± 0.05	0.403
NCI-H441 cells infected with *A. fumigatus* culture filtrates for 4 and 8 h										
	*SP-A1*		*SP-A2*		*SP-B*		*SP-C*		*SP-D*	
Time	Mean ± SE	*p*-value	Mean ± SE	*p*-value	Mean ± SE	*p*-value	Mean ± SE	*p*-value	Mean ± SE	*p*-value
4 h	0.76 ± 0.15	0.176	0.68 ± 0.18	0.137	0.59 ± 0.15	0.054	0.82 ± 0.03	*** <0.001	0.86 ± 0.26	0.619
8 h	0.74 ± 0.04	** 0.004	0.77 ± 0.08	* 0.046	0.69 ± 0.09	* 0.024	0.74 ± 0.05	*** <0.001	0.83 ± 0.05	* 0.022

* *p* ≤ 0.05, ** *p* ≤ 0.01, *** *p* ≤ 0.001.

## Data Availability

All data and materials used in the analysis are available upon request to the corresponding author.
